# Cancer vaccines as promising immuno-therapeutics: platforms and current progress

**DOI:** 10.1186/s13045-022-01247-x

**Published:** 2022-03-18

**Authors:** Jian Liu, Minyang Fu, Manni Wang, Dandan Wan, Yuquan Wei, Xiawei Wei

**Affiliations:** Laboratory of Aging Research and Cancer Drug Target, State Key Laboratory of Biotherapy, National Clinical Research Center for Geriatrics, West China Hospital, Sichuan University, No. 17, Block 3, Southern Renmin Road, Chengdu, 610041 Sichuan People’s Republic of China

**Keywords:** Cancer vaccine, Tumor antigens, Tumor resistance, Immunotherapy, Clinical application

## Abstract

Research on tumor immunotherapy has made tremendous progress in the past decades, with numerous studies entering the clinical evaluation. The cancer vaccine is considered a promising therapeutic strategy in the immunotherapy of solid tumors. Cancer vaccine stimulates anti-tumor immunity with tumor antigens, which could be delivered in the form of whole cells, peptides, nucleic acids, etc*.* Ideal cancer vaccines could overcome the immune suppression in tumors and induce both humoral immunity and cellular immunity. In this review, we introduced the working mechanism of cancer vaccines and summarized four platforms for cancer vaccine development. We also highlighted the clinical research progress of the cancer vaccines, especially focusing on their clinical application and therapeutic efficacy, which might hopefully facilitate the future design of the cancer vaccine.

## Background

The advent of vaccines has introduced new opportunities to prevent and treat infectious diseases. The earliest vaccine can be traced back to 1796 when Edward Jenner found that the cowpox vaccine protects against smallpox infection [1]. As the vaccine developed, it was later introduced to treat more diseases, such as cancers. The initial cancer vaccine based on tumor cells and tumor lysates was developed in 1980. Scientists used autologous tumor cells to treat colorectal cancer [2]. In the early 1990s, the first human tumor antigen melanoma-associated antigen 1 was identified [3], which opened a chapter on using tumor antigens in cancer vaccines. In 2010, a dendritic cell-based vaccine (Sipuleucel-T) was successfully used to treat prostate cancer, proving the viability of cancer vaccines and creating great excitement in the cancer vaccines field [4]. The outbreak of COVID-19 has urged the development of vaccine technology and brought cancer vaccines back into the public focus. Cancer vaccines mainly use tumor-associated antigens (TAAs) and tumor-specific antigens (TSAs) to activate the patient's immune system. Theoretically, the vaccine could provoke both specific cellular immunity and humoral immune response to prevent tumor growth and ultimately eradicate tumor cells [5]. Currently, most cancer vaccines are still in the stage of preclinical and clinical research [6]. More specific antigens and vaccine development platforms need to be developed.

The treatment purposes, which aim to kill tumor cells via tumor antigen-specific cellular immune responses, make cancer vaccines different from traditional vaccines. Only two FDA-approved prophylactic vaccines have been applied to prevent malignancies caused by viruses by now (Hepatitis B virus and human papillomavirus) [7]. Moreover, unlike traditional vaccines with antigens from foreign pathogens, tumor antigens are endogenous with low immunogenicity. Tumor antigens are often difficult to elicit an effective immune response [8]. Furthermore, traditional vaccines induce humoral immunity. However, CD8^+^ cytotoxic T cell-mediated cellular immunity is crucial in eliminating malignant cells for cancer vaccines [9].

Based on the different preparation methods, platforms for cancer vaccines are divided into four categories: cell-based vaccines, viruses-based vaccines, peptide-based vaccines, and nucleic acids-based vaccines [5]. Vaccines that use whole cells as antigen carriers are called cell-based cancer vaccines [10]. Cell-based vaccines are the main form of primeval cancer vaccines, where dendritic cells (DCs) vaccine has achieved significant results in clinical trials. Virus-based cancer vaccines mainly use viruses as vectors to treat and prevent tumors. Peptide-based vaccines are composed of known or predicted tumor antigen epitopes. Peptide-based vaccines are often less immunogenic, requiring a combination with adjuvants to enhance their immunogenicity [11]. Nucleic acid vaccines include DNA and RNA vaccines, composed of the encoding gene and carrier group of pathogen antigens. DNA cancer vaccines are closed circular DNA plasmids encoding TAAs or immunomodulatory molecules to induce tumor-specific responses [12]. mRNA vaccines are synthesized in vitro, they could encode antigens and express proteins following internalization to stimulate an immune response [13]. In recent years, combining cancer vaccines with various immunotherapies or standardized treatments has become an effective strategy for overcoming tumor resistance and improving clinical outcomes.

Cancer vaccines have been intensively studied over the past decade. The availability and low cost of high-throughput sequencing technologies have led to the identification of numerous tumor neoantigens. The in-depth study of immunological mechanisms and various new vaccine platforms have extensively promoted cancer vaccines research. In this review, we introduced the working mechanism of cancer vaccines and summarized four platforms for the development of cancer vaccines. We also highlighted the research progress of the cancer vaccine, especially focusing on their clinical application and therapeutic efficacy, which might hopefully facilitate the future design of the cancer vaccine.

## Mechanism of cancer vaccines

### Tumor antigens

Antigen selection is a critical process of cancer vaccines design. Tumor antigens recognized by T lymphocytes are central to the efficacy of cancer vaccines [14]. The ideal antigen for a cancer vaccine should be highly immunogenic, explicitly expressed in all cancer cells (not in normal cells) and necessary for the survival of cancer cells [15]. Tumor antigens can be divided into TAAs and TSAs. TAAs also be known as tumor-shared antigens. TAAs include “self-antigens” such as differentiated antigens, overexpressed antigens, cancer-testicular antigens, and viral-original “non-self” antigens [6]. Prominent examples of overexpressed tumor antigens are human epidermal growth factor receptor 2 (HER2) and human telomerase reverse transcriptase [16]. Tissue differentiation antigens are expressed by tumor cells and normal cells of the same tissue origin as tumor cells, such as prostate-specific antigen (PSA) expressed in the prostate gland and prostate cancer [6], melanoma antigens tyrosinase expressed by normal melanocytes, and melanoma cells [17]. TAAs are adaptable and can be applied to different patients. Early cancer vaccines were primarily focused on TAAs. However, due to the central immune tolerance of the thymus, activated T cells that recognize TAAs or other autoantigens may be eliminated during development, which will affect the efficacy of the vaccine [18]. Thus, cancer vaccines that use TAAs must be compelling enough to “break the tolerance.” Although TAAs have been focused on for many years, clinical trials of cancer vaccines based on TAAs have had limited success [19]. In addition, TAAs are also expressed in non-malignant tissues, increasing the risk of vaccine-induced autoimmune toxicity.

TSAs are a class of proteins specifically expressed in tumor cells. TSAs are mentioned as neoantigens sometimes. The individual-specific non-autogenous proteins produced due to mutations in tumor cells are called neoantigens [20]. Neoantigens are expressed only by tumor cells, triggering a valid tumor-specific T-cell response with limited “off-target” damage [21]. Compared with TAAs, neoantigens have more potent immunogenicity and higher major histocompatibility complex (MHC) affinity. What’s more, they are unaffected by central immune tolerance [22]. The wide application of next-generation sequencing technology makes it possible to identify personalized neoantigens in a timely and cost-effective manner. Further, the development of algorithms for predicting MHC I class binding epitopes has also greatly facilitated the discovery of potential new immunogenicity epitopes [23]. Cancer vaccines targeting neoantigen have become the main direction of tumor vaccine in recent years. Recently, several clinical trials evaluating neoantigen vaccines have yielded promising results with improved patient survival [24]. An mRNA neoantigen melanoma vaccine is a typical example that induced T cell infiltration and neoantigen-specific killing of autologous tumor cells [25]. The incidence of metastatic events was significantly reduced after vaccination, resulting in sustained progression-free survival [25]. Besides, the neoantigen-loaded DC vaccination could provoke a T cell-specific response that led to antigen spreading in patients with melanoma [26]. Although personalized cancer vaccines based on neoantigens have shown encouraging results, a large number of predicted neoantigens tend to trigger very few actual anti-tumor responses [27]. In addition, differences between tumor types and individuals limit the application of mutated neoantigens-targeted cancer vaccines. Therefore, it is crucial to find neoantigens with good quality for the development of neoantigen vaccines.

The high-quality neoantigens should be associated with the following features: First, they should manifest strong binding affinity to human leukocyte antigen (HLA); second, they should be highly heterologous compared to the wild type; third, they can be expressed by most tumor cells; fourth, they are generated as the consequences of mutations that affect survival. The neoantigens with these features could induce a robust immune response and prevent the development of tumor-immune escape [28]. Currently, no studies have shown the optimal number of neoantigens for a tumor vaccine. A neoantigen vaccine usually contains several to dozens of neoantigens. For example, a personalized neoantigen DNA vaccine (GNOS-PV02) encodes up to 40 neoantigens, including all detected neoantigens for the majority of hepatocellular carcinoma patients [29]. In recent years, to increase the vaccine's effectiveness, scientists have combined shared antigens with neoantigens to expand the antigen pool for vaccination. For example, the APVAC1/2 vaccines, which contain shared tumor antigens and patient-specific neoantigen, can effectively activate the T-cell response in the treatment of glioblastoma [30]. Furthermore, early clinical studies of personalized neoantigen vaccines combined with PD-1 or PD-L1 inhibitors have also shown anti-tumor activity [31, 32].

### Stimulation of anti-tumor immunity

Antigen-presenting cells (APCs) are crucial in immune activation induced by tumor antigens. DCs, the critical bridge connecting innate immunity and adaptive immunity, are the most important ones. DCs are initial antigen presenters that could take up antigens and cross-present them on MHC I molecules [33]. Immature DCs have a strong ability to recognize and capture antigens through phagocytosis and micropinocytosis. The activation of immature DCs by Toll-like receptor ligands in the tumor microenvironment (TME) could temporarily enhance antigen-specific micropinocytosis [34], which potentially increases the ability of DCs to capture antigens with toll-like receptor ligand adjuvants. After antigen uptake, MHC I, MHC II, and costimulatory molecules on the surface of DCs will be upregulated, and they gradually lose their ability to take up antigens [35]. The antigen-loaded DCs migrate to draining lymph nodes, which are the primary site of T cell priming.

Mature DCs present the processed antigen epitopes on MHC I and MHC II molecules to naive CD4^+^ and CD8^+^ T cells [36]. Moreover, DCs also secrete IL-12 and interferon-*γ* (IFN-*γ*) to increase costimulatory factor production [37]. Tumor-specific T cells are activated by binding to MHC–peptide complex–T cell receptor and costimulatory “signal 2”. Activated T cells then differentiate into long-lived memory T cells and effector T cells. Effector tumor-specific T cells amplify and are trafficked to TME to induce tumor killing through cytotoxicity and the production of effector cytokines [38]. In addition, activated B cells promote tumor apoptosis through antibody-dependent cellular cytotoxicity (ADCC) or complement-dependent cytotoxicity [39]. Further, immunogenic cell death release tumor antigens and damage-associated molecular patterns [40]. In turn, the tumor antigens released by lysed tumor cells can be captured, processed, and presented again by APCs to induce polyclonal T cell responses, thereby increasing the antigenic breadth of anti-tumor-immune responses [41]. These processes are known as the cancer-immunity cycle [42].

**Fig. 1:**
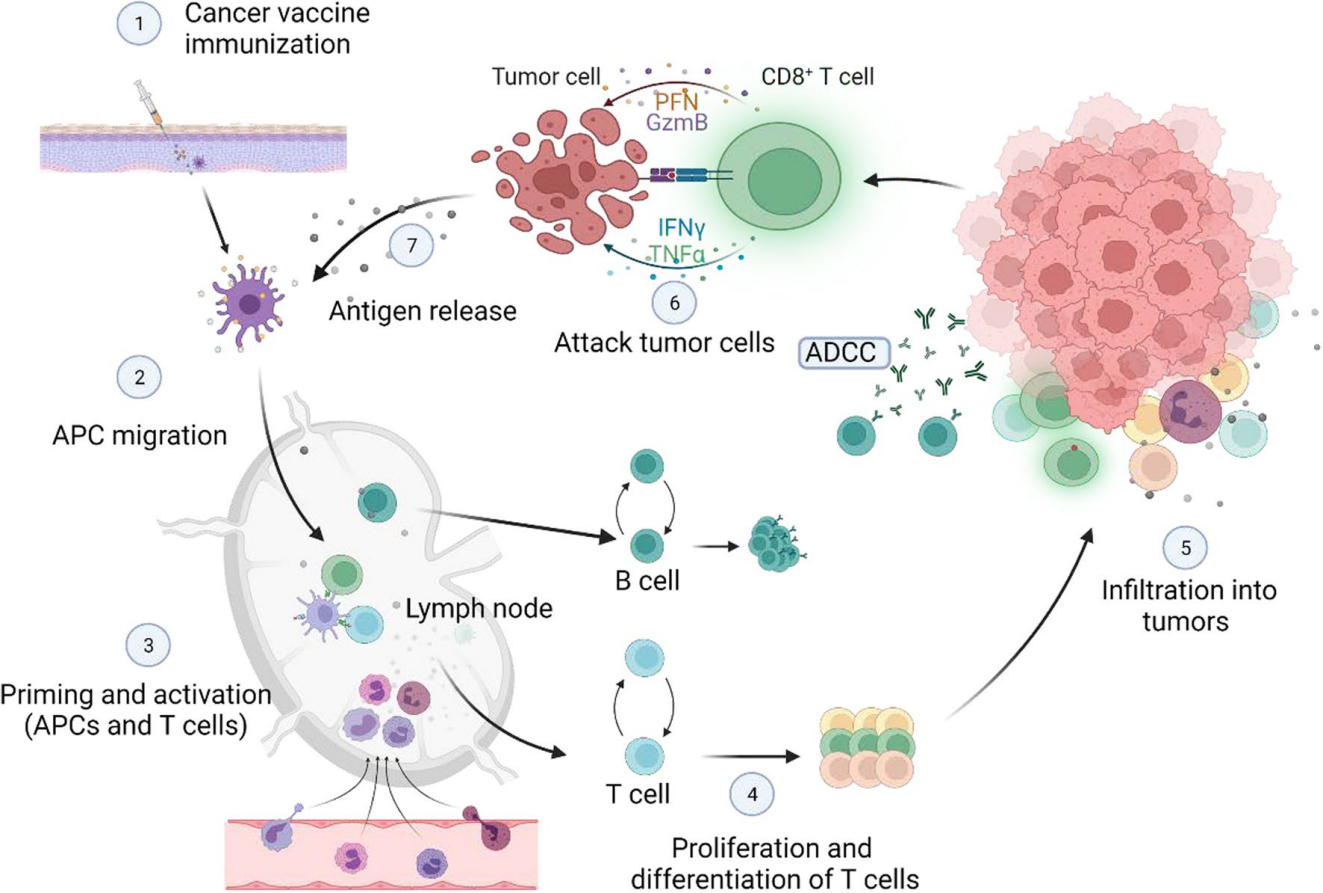
Tumor-immune cycle induced by cancer vaccines. The immune response that effectively kills tumor cells involves steps that allow repetition and expansion called the tumor-immune cycle. After the administration of the tumor vaccine, DCs uptake and process tumor antigens, then present them to MHC II or MHC I (through cross-presentation). Antigen-loaded DCs migrate to lymph nodes to recruit and activate immune cells. Follicular DCs promote the generation of memory B cells and plasma cells. Activated B cells promote tumor apoptosis through ADCC. Activated T cells proliferate and differentiate into memory T cells and effector T cells. Effector T cells travel to TME, killing tumor cells directly or inducing tumor cell apoptosis. Immunogenic dead tumor cells can release TAAs and danger signaling molecules to increase the depth and breadth of the response in subsequent cycles

CD4^+^ T cells work in collaboration with various immune cells. CD4^+^ T cells trigger continuous T cell initiation, expansion and antigen spread, thus expanding the anti-tumor T cell repertoire [43]. IFN-*γ* secreted by Th1 CD4^+^ T cells upregulates MHC I on tumor cells, improving the killing effector of CD8^+^ T cells. Furthermore, Th1 CD4^+^ T cells promote the inflammatory microenvironment by acting on various immune cells in tumors. CD4^+^ T cells also control the differentiation of CD8^+^ T effector cells. Cytotoxic T lymphocytes (CTLs) are crucial cells for killing tumor cells and presenting their cognate antigen [44]. After antigen receptor-mediated activation, CD8^+^ T cells proliferate and differentiate into effector cells called CTLs. Activated CTLs will penetrate the core of the tumor or infiltrate the site to kill tumor cells. The number of CTLs in TME is a critical prognostic marker of cancer. CTLs detect tumor cells presenting target antigens and attack target cells through different mechanisms [45]. First, CTLs could kill cancer cells by producing and releasing cytotoxic particles such as perforin and granzymes. Furthermore, CTLs induce apoptosis of target cells through Fas ligand (FasL)-mediated interactions [45]. In addition, the release of IFN-*γ* and tumor necrosis factor *α* (TNF-*α*) by CTLs induces cytotoxicity of cancer cells [46]. IFN-*γ* could inhibit the angiogenesis of cancer cells and cause macrophage polarity to M1 cells. IFN-*γ* produced by CTLs supports their further differentiation into effector CTLs [47]. In summary, cancer vaccines eradicate tumor cells mainly by activating cellular immunity, and cancer vaccines start the cancer-immunity cycle to play a persistent anti-tumor role (Fig. 1).

### The barriers in vaccine therapy: Immune resistance

**Fig. 2:**
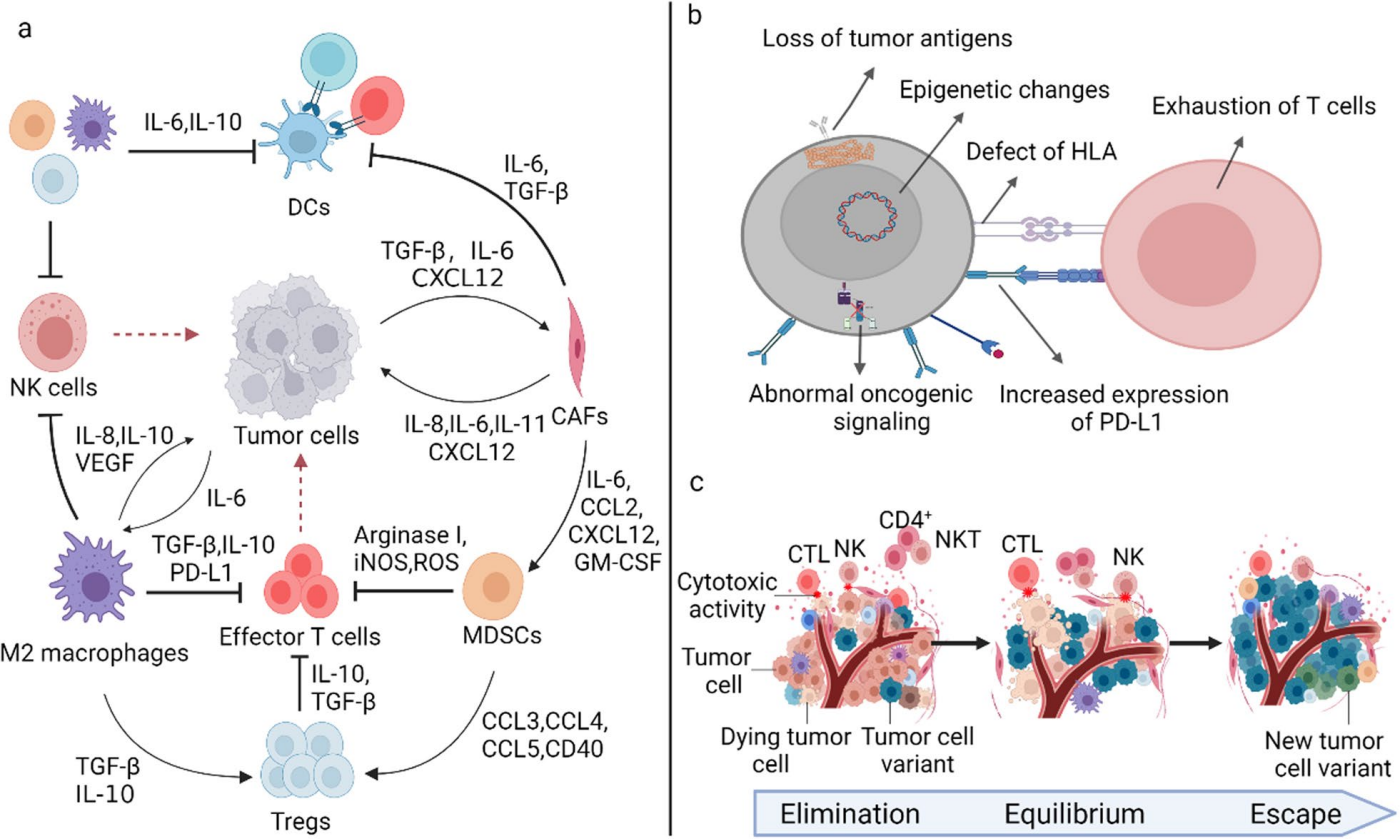
Resistance of cancer vaccines. **a** Tumor external resistance. Immunosuppressive cells (such as CAFs, MDSCs, Tregs, and M2 macrophages) and immunosuppressive cytokines can inhibit the activation of effector T cells and DC-mediated T cells directly or indirectly in TME. **b** Tumor intrinsic resistance. The intrinsic resistance of tumor contains six aspects: the mutations in signaling pathways supporting tumor-immune control; the loss of tumor antigen expression; the changes in antigen processing pathways; the loss of HLA expression; epigenetic changes; increased expression of immunosuppressive ligands **c** Immune selection: from immunosurveillance to tumor escape

Tumor-immune escape can be divided into intrinsic mechanisms determined by the characteristics of tumor cells and external mechanisms involving tumor matrix components (Fig. 2). These two mechanisms determine the efficiency of cancer vaccines.

#### Tumor intrinsic resistance

The resistance to cancer vaccines derives from intrinsic factors, including mutations in signaling pathways supporting tumor-immune control, downregulation or lost tumor antigen expression, altered antigen processing pathways, or loss of HLA expression. Above all could result in poor recognition of tumor cells by T cells [48]. The depletion of tumor antigens is a potential immune escape mechanism, especially for tumor cells whose survival could not be determined by the depleted antigens [49]. The deletion of tumor antigens can be attributed to copying number loss at the genomic level or epigenetic changes. In addition, antigens loss also is mediated by immune selection. Due to differences in immunogenicity among tumor cells, those tumors with strong immunogenicity induce an effective anti-tumor-immune response and will be eliminated by the body. Tumors with relatively weak immunogenicity can evade the immune system and selectively multiply, called immune selection. With continuous selection, the immunogenicity of the tumor becomes weaker and weaker. Additionally, low expression of HLA molecules and lacking presentation function are often lead to tumor-immune escape. HLA plays many roles in antigen processing and presentation. Tumor cells can avoid T cell killing by downregulating the expression of HLA on the surface. Lack of costimulatory molecules is also a cause of tumor escape. The absence of costimulatory signals (such as B7, CD40, CD28) could cause the activation of T cells to fail and cause T cell tolerance. Furthermore, changes in tumor intrinsic signaling pathways may also cause immune escape [50]. For instance, WNT/β-catenin signaling pathway abnormal activation was associated with tumors that lack immune cell infiltration and are less likely to respond to immune checkpoint blockade [51].

#### Tumor extrinsic resistance

The extrinsic resistance of cancer vaccines may be caused by immunosuppressive cells in the immune microenvironment, including myeloid-derived suppressor cells (MDSCs) [52], tumor-associated macrophages (TAMs) [53], T regulatory cells (Tregs) [54], protumor N2 neutrophils, and cancer-associated fibroblasts (CAFs). Immunosuppressive cells interfere with the activation and proliferation of T cells by upregulating the expression of immunosuppressive receptors (such as PD1 or CTLA-4) and secreting immunosuppressive cytokines (such as IL-6, IL-10, TGFβ, and VEGF) [55, 56]. In addition, immunosuppressive cells can inhibit DC's function, promoting tumor resistance. MDSCs are pathologically activated neutrophils and monocytes with strong immunosuppressive activity [57]. MDSCs are the cornerstone of the immunosuppressive barrier protecting tumors from the patient's immune system and immunotherapy [58]. CAFs are the key component of the TME. CAFs can prevent the proliferation and migration of DC, recruit MDSCs, and inhibit T cell invasion by remodeling the extracellular matrix to construct dense fibrous stroma [59]. TAMs are classified as anti-tumorigenesis M1 (classically activated) and pro-tumorigenesis M2 (alternatively activated) phenotypes [60]. TAMs were polarized into M2 macrophage by Th2 cytokines (IL-4, IL-10, TGFβ1) and immunocomplexes [61]. M2 phenotype macrophages could promote the evolution of tumor-related vasculature assist tumor cells in acquiring activation and remodeling stromal features to support tumors [62].

In summary, the mechanisms of tumors resistance to vaccines are multifaceted and complex, which means we need multiple approaches to overcome resistance. Different modalities can be used in combination depending on the particular resistance mechanism in the patient population. Several strategies have been developed to solve tumor escape and TME immunosuppression, including improving immunotherapy delivery platforms and improving antigen selection and combination therapy. Radiotherapy, chemotherapeutic agents, and immunomodulatory molecules may work synergistically with cancer vaccines [6].

## Cancer vaccines platforms

**Fig. 3:**
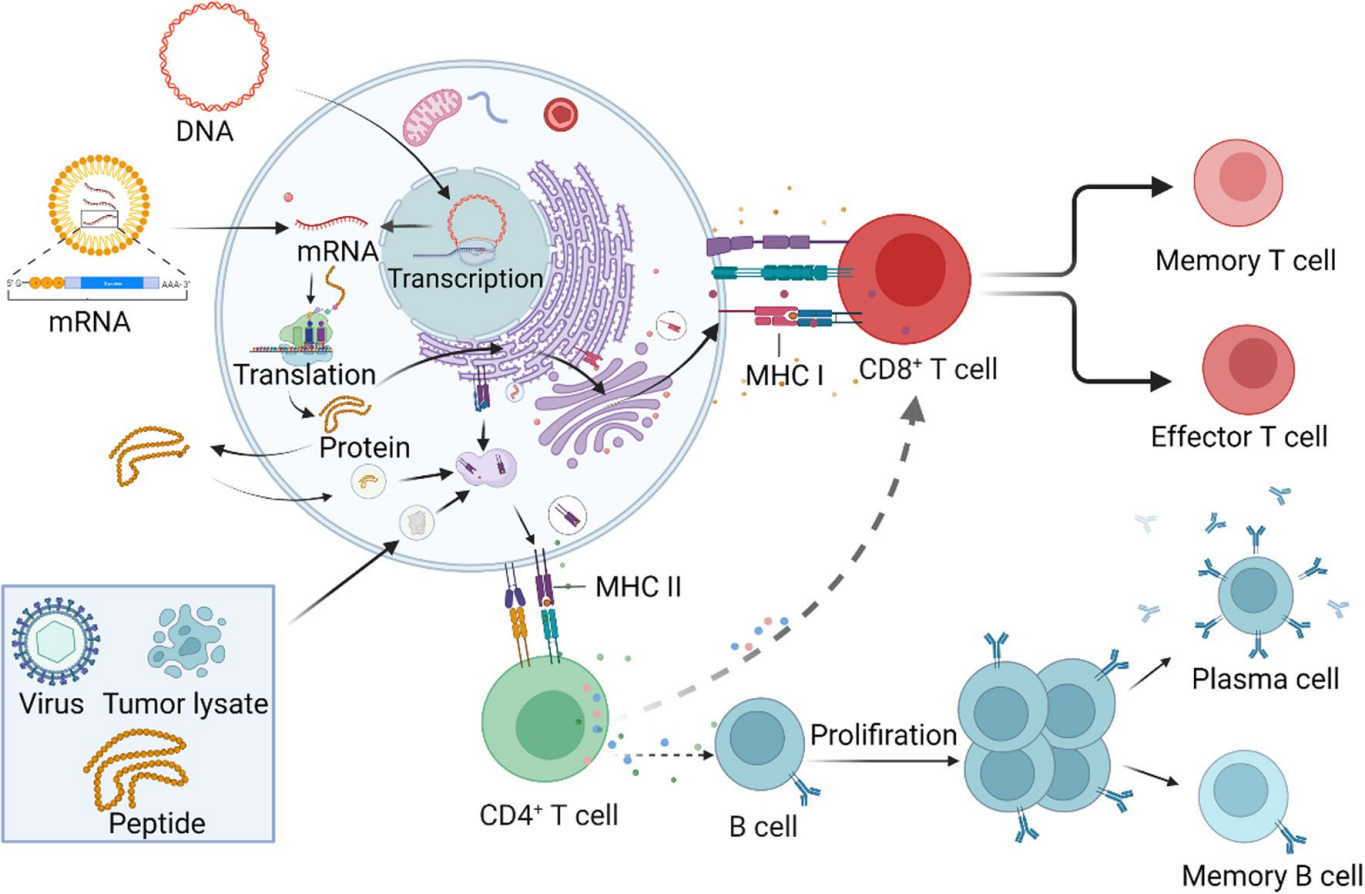
Mechanism of cancer vaccines. Compared to peptide-based vaccines, nucleic acid-based vaccines need more processing steps after entering the body before being presented to T cells by DCs. However, DNA and RNA vaccines are better suited to deliver MHC I presentation antigens than peptide vaccines. Tumor antigens are processed by DCs and transported to the cell surface of MHC I and MHC II molecules. Interaction between MHC–peptide complex–T cell receptor (TCR) and cognate receptor-ligand pairs activate T cells. Activated CD4^+^ T cells induce B cells to differentiate into plasma cells and memory B cells. Activated T cells differentiated into CD8^+^ memory T cells and CD8^+^ effector T cells. Eventually, effector T cells, B cells, antibodies, and some cytokines kill tumor cells directly or indirectly

Cancer vaccines can be divided into four categories: cell-based vaccines, peptide-based vaccines, viral-based vaccines, and nucleic acid-based vaccines (Fig. 3). Cell-based vaccines are the form of cancer vaccines initially. Cell-based cancer vaccines are often prepared from whole cells or cell fragments, containing almost tumor antigens, inducing a broader antigen immune response. DC vaccine is an important branch of cell-based vaccines. Personalized neoantigen cancer vaccines based on DC have shown promising anti-tumor effects in clinical. However, the cumbersome process and expensive cost limit the development of DC vaccines. Viruses are naturally immunogenic and their genetic material can be engineered to contain sequences encoding tumor antigens. Several recombinant viruses, such as adenovirus, can infect immune cells as vectors. The engineered virus vaccines can present tumor antigens in large quantities in the immune system and produce anti-tumor immunity. Furthermore, the oncolytic virus can be used as a vector as well. Except for providing tumor antigens, the virus itself can also lyse the tumor, release tumor antigens, further increase the vaccine's effectiveness, and produce long-term immune memory. However, the vaccine production process based on the viral vector is complex.

Peptide-based subunit vaccines, including chemical and biosynthetic preparations of predicted or known specific tumor antigens, induce a robust immune response against the particular tumor antigen site. Peptide-based subunit vaccine combined with adjuvants can efficiently provoke humoral immune response, suitable for preventing and treating viral infectious diseases. HBV and HPV vaccines for liver and cervical cancers were primarily peptide-based subunit vaccines. Especially, virus-like particles (VLP)-based subunit vaccines that can activate cellular immune responses have shown good anti-tumor activity in recent years. The nucleic acid vaccine is a promising vaccine platform. The nucleic acid vaccine induces strong MHC I mediated CD8 + T cell responses; thus, it is a desirable cancer vaccine platform [63]. Nucleic acid vaccines can simultaneously deliver multiple antigens to trigger humoral and cellular immunity. Additionally, nucleic acid vaccines can encode full-length tumor antigens, allowing APC to cross-present various epitopes or present several antigens simultaneously. Finally, the nucleic acid vaccine preparation is simple and fast, which is suitable for developing personalized neoantigen cancer vaccines.

Various factors should be considered when selecting a vaccine platform. The time required to prepare individualized vaccines is critical in determining vaccine platforms. For some metastatic diseases, the nucleic acid vaccines would be the best choice to save time. During the preparation of vaccines, some combination therapies could also be used to alleviate further disease exacerbations and cultivate a favorable immune environment to enhance the immune response. In addition, the route of administration and the number of vaccinations should be considered when we select a vaccine preparation platform. In addition to the choice of platform, the optimization and design of antigens are also critical. Tumor antigen coupling binding vectors (e.g., tetanus endotoxin, diphtheria toxoid) can improve antigen immunogenicity. Antigen-optimized design based on protein structure, such as virus-like particles vaccines, can enhance the immune response. In addition, the use of bioinformatics and deep sequencing techniques to guide vaccine design is necessary. Here we sought to summarize the latest optimization strategies of four types of cancer vaccines, especially nucleic acid vaccines.

### Cell-based cancer vaccines

The cell-based vaccine can be divided into tumor cell vaccine and immune cell vaccine. The whole tumor cell vaccine is a relatively simple and direct approach to tumor immunotherapy. The tumor cell vaccine contains the whole tumor-associated antigens, including the epitopes of CD4^+^ helper T cells and CTLs. The immune cell vaccine is based on cells' role in the immune system. DCs are the most powerful professional APCs in the body. In most cases, DCs are needed to present cancer antigens in vaccination. Hence, it is an effective way for tumor immunity to import tumor-associated antigens into DCs to make them play the role of antigen presentation and activate T cells. Most DC vaccines are sourced from monocyte-derived DCs (MoDCs) [64]. In the immunotherapy study, tumor cell lysate was loaded in MoDCs, and the MoDC-based vaccination revealed good tolerance and efficacy [65]. The focus of DC-based vaccines was not only DC cells themselves; the exosomes released by DCs (DCexos) were paid attention. DCexos are inert membrane vesicles with biostability, which can express MHCI, MHC II, and costimulatory molecules [66]. DCexos have already shown efficacy in treating cancer in clinical trials. However, DCexos have failed to demonstrate obvious clinical benefit yet [67, 68].

#### Current progress in cell-based cancer vaccines

It is critical for the whole tumor cell vaccine to improve its immunogenicity. Live tumor cells showed poor immunogenicity due to the secretion of soluble factors which could suppress immune cells [69]. Therefore, certain measures were often taken to increase the immunogenicity of tumor cells and improve the efficacy of whole tumor cell vaccines. For example, dead cells induce an immune response better than living cells. The death of tumor cells could provoke an adaptive immune response [70]. The way to induce immunogenic cell death (ICD) is various. It has been reported that compared to replication-deficient adenovirus or oxaliplatin, oncolytic adenovirus had better inducement of ICD [71]. Using interferon genes (STING)-activating nanoparticles to induce the apoptosis of neuroblastoma cells could also increase the immunogenicity [72]. Moreover, photosens (PS)-induced dead tumor cells and RAS selective lethal 3-induced ferroptosis tumor cells could similarly show strong immunogenicity [73, 74].

In addition, the modification of tumor cells could also improve the efficacy of whole tumor cell vaccines. In most cases, the purpose of the modification is to enhance the presentation of antigen, which means the immune response-related molecules could take part in the modification. For instance, IL-21 and IL-7 are two crucial factors that could synergistically strengthen T cell response. The whole tumor cell vaccines with the genetic modification of IL-21 and IL-7 illustrated great efficacy [75]. Double-transfecting IL-15, a modulator NK and memory T cells, and its truncated receptor IL-15R*α* into CT26 cells induced a robust anti-tumor response as well [76]. Besides, some adjuvant decoration approaches have been used to modify the vaccine, like the dying tumor cells decorated by CpG-loaded nanoparticles were found to promote antigen presentation [77]. Finally, combining the whole tumor cell vaccine and immune checkpoint inhibitors (CPI) is more common. It is intended to block the pathways that suppress self-reactive T cells' activity. It has already been proved that the block of programmed cell death ligand 1 (PD-L1) and cytotoxic T-lymphocyte-associated protein 4 (CTLA-4) can enhance the cell-based therapeutic vaccination [78, 79].

Optimizing immune cell vaccines, especially DC vaccines, could involve more parts. The first one is the sort of DC. MoDCs, the most commonly used DC, were flawed because of the time of generation and function limitation. Conversely, naturally occurring DCs have the greater capability of presenting antigens because they express higher MHC molecules. It has been proved that the efficacy of the DC vaccine could be influenced by the type of DC [37]. The common alternatives to MoDCs are conventional DCs (cDCs) and myeloid/plasmacytoid DCs (mDCs/pDCs). In two clinical trials, natural dendritic cells, including mDCs, cDCs, or a combination of both, were used to treat patients with chemo-naive metastatic castration-resistant prostate cancer and stage III melanoma (NCT02692976, NCT02574377) [80]. mDCs and cDCs are functionally different because they have distinct Toll-like receptors (TLRs) and cytokines secretion. Therefore, their combined use might be synergistic, confirmed in treating murine lung cancer [81]. The antigen-loaded by DC cells usually comes from tumor lysates or tumor-derived mRNA, specific TAA-based peptides or TAA-coding mRNA and even whole tumor (fusion of DCs with tumor cells) [82]. Furtherly, some neoantigens which could enhance immune response and decrease autoimmunity risk were evaluated in DC-based vaccines. It may promote the development of the personalized treatment of DC vaccines [83, 84].

Radiotherapy (RT) is another widely used optimizing strategy. DC vaccines can be combined with RT to increase the immune response. The radiotherapy could cause the recruitment of RT-recruited tumor-associated neutrophils (RT-Ns), resulting in the increase of reactive oxygen species (ROS) and the oxidative damage of tumor cells [85]. With the apoptosis of tumor cells, the tumor neoantigens and danger signal would be released, promoting the recognition of antigens and maturation of DCs [86]. In addition, to further improve the efficacy, the DC vaccine could be used with the combination of adjuvants, cytokines, immune CPI, or chemotherapy [87].

### Virus-based cancer vaccines

One of the primary benefits of virus-based vaccines is that the vaccine can make innate and adaptive immune work together to achieve an effective and long-lasting immune response. The virus-based vaccine can be divided into three forms: inactivated, live attenuated, or subunit vaccines against the virus that can cause the tumor; the oncolytic virus vaccine and the virus vector vaccine. It was reported that about 12% of cancer is attributed to viral infections. Epstein-Barr virus, HBV, Hepatitis C virus, and HPV are the most common cancer-related viruses [88]. Inactivated whole virus vaccines have shown promising efficacy in treating Covid-19 or Ebola [89, 90]. Logically, it would show the same effectiveness in treating virus-related cancer [91]. However, it was used less frequently in oncological diseases, probably because of difficulties in production and safety issues. Instead, with the development of bioengineering technology, the approach like virus-like particle is increasingly used in the treatment [92, 93].

Oncolytic virus is a novel immunotherapy that explicitly kills tumor cells and promotes anti-tumor responses. After being infected by the oncolytic virus, tumor cells produce ROS and cytokines, stimulating immune cells. Subsequently, the oncolysis would happen, and substances like TAAs would release as well [94]. The anti-tumor efficacy of oncolytic viruses has already been proved in various clinical trials. The sorts of oncolytic virus contain herpes simplex virus, adenovirus, measles, vaccinia, reovirus, vesicular stomatitis virus, etc. [95]. Among them, T-VEC, a first-generation recombinant herpes simplex virus product, has been the most striking one so far [96]. In addition to the herpes simplex virus, the adenovirus is another commonly used oncolytic virus, which is frequently used as delivery vectors for specific genes as well [97]. Adenovirus is easy to manipulate, its gene structure is clear, and it is easy to achieve gene transfer and tumor antigen expression. Second, adenoviruses have a very broad spectrum of host cell tropism and can be rapidly prepared in large quantities. In addition, mucosal infection is an inherent feature of adenoviruses. Therefore, the adenovirus vector is a promising vaccine platform [98]. Adenovirus-based cancer vaccines, both as non-replicating vectors and oncolytic adenoviruses, have shown promise in preclinical and clinical trials [99]. Except for adenovirus, other vectors like vaccinia virus, lentivirus adeno-associated virus are also used on the tumor vaccine platform, in which lentivirus and adeno-associated virus have the unique ability to stably and long-termly express the transgene in non-dividing cells the same as adenovirus [100–102].

#### Optimization strategy for virus-based cancer vaccines

Improving TME is a viable strategy to optimize the efficacy of virus-based vaccines. With the increasing knowledge on the mechanisms of immunosuppression in TME, various strategies combined with viral vaccines have become feasible. Combining virus-based vaccines with PD-1 inhibitors is the most common. The vaccination of virus vaccine combination with PD1 blockade showed a long-term tumor-free survival in the tumor model [214]. Except for combining with checkpoint inhibitors, other potential ways should be concerned. Widely studied protein YAP, a coactivator of the Hippo pathway, was found to do with Treg regulation, which is a significant immunosuppressive cell. The deficiency of YAP would cause the dysfunction of Treg. Hence, targeting YAP could improve the cancer vaccine's anti-tumor efficacy by destroying the immunosuppressive environment made by Tregs [103]. In addition, tissue growth factor β (TGF-β) was also found to induce immune suppression by influencing multiple immune cells, including T cells and NK cells [104]. So, TGF-β might also be a great target to decrease tumor-induced immunosuppression. It has already been proved that M7824, a novel bifunctional anti-PD-L1 and TGF-β, could synergistically increase the efficacy of virus-based cancer vaccines [105].

In addition to combination therapy, virus-based vaccines are also designed to express immune-regulating molecules to disrupt TME. Patients receiving TG4010, a therapeutic viral vaccine encodes human mucin-1 and interleukin 2, have shown a longer median survival period [106]. BT-001, another oncolytic virus vaccine, which could express anti-CTLA4 antibody and granulocyte macrophage colony stimulating factor (GM-CSF), is worthy of being concentrated on. It has already been tested in a clinical trial (NCT04725331). Besides, other strategies like the fusion-enhanced oncolytic immunotherapies based on the simplex virus, as well as the combination of virus-based vaccine to chemotherapy, adoptive T cell therapy and radiation therapy, are being further tied [107]. The antiviral immune response neutralizing the viral vector needs to be avoided for the virus vectors vaccine. Heterogeneous priming and enhancement strategies mean using one viral vector to deliver the antigen and then using different types of viral vectors to deliver the same tumor antigen for enhancement [15]. A typical example is PROSTVAC-V/F, which uses PSA-encoded vaccinia virus as a primary immunization vaccine and PSA-encoded avian poxvirus as a booster vaccine [108].

### Peptide-based cancer vaccines

The current focus of cancer vaccines has shifted from complete, inactivated, or attenuated pathogens to vaccines based on subunit components. Peptide-based vaccines are polypeptides composed of known or predicted tumor antigen epitopes. Due to the limitation of MHC polymorphism and the small size of antigen epitopes themselves, the immunogenicity of peptide-based vaccines is weak. It is often difficult to cause a robust immune response, which also leads to immune tolerance. Adjuvants are combined with peptide-based vaccines to enhance the immune response generally. Not all regions of protein antigens are equally immunogenic for B cell and T cell. Compared to inactivated tumor cell vaccines, peptide-based vaccines trigger a more focused immune response against critical neutralization epitopes. This advantage in immunity is called immunodominance [109]. Peptide-based cancer vaccines usually require both CD8^+^ T epitopes and CD4^+^ T cell epitopes. CD8^+^ T cell epitopes activate CTLs tumor immunity through the antigens cross-presentation pathway, while CD4^+^ T cells activate helper T cells to maintain the function of CTLs [110].

The length of the peptide chain largely determines the performance of the peptide vaccine. Short peptides are usually the smallest CD8^+^ T cell epitope and have a short half-life in vivo. This peptide does not require processing in specialized APCs and is directly loaded onto the MHC I molecules of APCs or other nucleus cells. Lacking costimulatory molecules required for optimal CD8^+^ T cell activation limits the provoking of CTLs [111]. Therefore, short peptides often temporarily activate CTLs and even induce CTLs tolerance [112]. Furthermore, shorter peptides also tend to be limited by HLA types. Compared to short peptides, long peptides allow broader coverage of HLA containing multiple epitopes while also can support the recognition and binding of motifs to enhance immunogenicity. Long peptides must be processed by APCs rather than directly loaded to MHC molecules [113]. After internalization, a part of the long peptides is degraded by the endosomal pathway, loaded onto MHC-II molecules, and then recognized by CD4^+^ T helper cells. The other parts enter the cytoplasmic or vacuolar pathway and are cross-presented by MHC-I molecules to activate CD8^+^ T cells [49]. Thus, long peptide vaccines are more potential to induce sustained and effective anti-tumor activity responses. Short peptides are usually produced by chemical synthesis, while long peptides are frequently produced by protein expression systems. The immunogenicity varies among recombinant protein subunit vaccines based on different expression platforms. Several expression platforms have been used for the production of cancer vaccines, including Escherichia coli (E.coli) [114], plants, yeasts [115], insect cells, and mammalian cells. The proteins expressed by mammalian cells are most closely to natural tumor antigens. Baculovirus-insect cell is a potential system with low cost and some post-expression modification of target proteins [116].

#### Improving the efficacy of peptide-based cancer vaccines

Small antigens can enhance their immunogenicity by fusing with carrier proteins, such as heat shock proteins (HSP) and keyhole limpet hemocyanin (KLH). Jiang et al. found that mice immunized with the MAGE-1-Hsp70 fusion protein caused significantly higher titers of melanoma antigen (MAGE-1) specific antibodies, more IFN-*γ* secretion and stronger cytotoxic T lymphocyte activity than mice immunized with MAGE-1 alone [117]. Fusion cancer vaccines based on HSP may enhance the anti-tumor-immune response by improving the ability of APCs to present tumor antigen peptides on their surfaces through the MHC I for identification by CD8^+^ T cells. However, current clinical vaccine trials on the HSP complex have had little success [118, 119]. Furthermore, smaller antigen epitopes also are taken in repeated series or connected to other different antigen epitopes to enhance the immunogenicity of the vaccine [120]. For example, the MUC1 cancer vaccine, which fuses tetanus toxins, significantly inhibits breast tumors' progression by preventive vaccination in mouse models [121]. Cancer vaccines fused with different antigen epitopes deserve further study.

Furthermore, VLPs have emerged as a potential peptide-based vaccines platform since VLPs can enhance lymph node transportation and phagocytosis of antigens. VLPs enrich epitopes on the surface and significantly enhance the activation of humoral immunity. VLPs have been widely used in developing influenza vaccines and cancer vaccines in recent years. VLPs demonstrate a specific interaction between antigens and pattern recognition receptors (PRRs) of DCs, a process similar to recognizing natural virus particles that can effectively activate immunity [122]. Typical VLPs include hepatitis B virus core antigen (HBcAg), ferritin, bacteriophage P22, bacteriophage Qβ. The core protein of HBcAg is a highly immunogenic VLP. Scientists have demonstrated that chimeric claudin18.2 HBcAg nanoparticle vaccine could elicit auto-antibodies with high cytocidal and tumoricidal potential [123]. A recent study showed that the chimeric melanoma antigen HBcAg vaccine could significantly inhibit melanoma growth and metastasis in animal studies [124]. Ferritin is another kind of VLP composed of 24 self-assembled subunits, and its unique hollow spherical cage-like symmetric structure is suitable for displaying various pathogenic-associated antigens [125]. Scientists recently developed a ferritin nanoparticle vaccine against chronic hepatitis B using “SpyTag-SpyCatcher” and in vitro spontaneous peptide bond formation system [126, 127]. Besides, some other VLPs like bacteriophage Qβ were used to display MUC-1. It induced the production of highly potent MUC-1-specific antibodies and CTLs [128].

Peptide-based vaccines are often used in combination with adjuvants. Adjuvants largely determine the type and extent of T cell response after vaccination. Cancer vaccines aim to induce the activation and proliferation of CTLs, which require Th1-type immune response dominated by IFN-*γ*. Several novel adjuvants show tremendous potential for anti-tumor effects, including nanomaterials or synthetic TLRs ligands and cytokines. These adjuvants are widely used in the research of various cancer vaccines, including nucleic acid-based vaccines and cell-based vaccines. Theoretically, these new adjuvants contribute to the recruitment of leukocytes to the vaccination site, support the expansion and activation of T cells, and promote their migration to lymph nodes and tumor sites [11]. Aluminum adjuvants significantly promote Th2-type responses, making them less suitable for cancer vaccines [11]. Conversely, AS04, an aluminum salt in combination with MPL (a detoxified form of lipopolysaccharide), can provoke a prominent Th1-type response, producing INF-*γ* [129]. Incomplete Freund's adjuvant (IFA) is the most commonly used in human cancer patients. Clinical-grade IFA has been widely used in clinical research of various protein-based cancer vaccines [130]. Additionally, nanomaterials are widely studied as attractive antigen-delivery systems because they protect proteins from rapid degradation by proteases, thereby increasing the half-life of antigens. They can be engineered to target specific types of cells and organs. A classic example is the use of polyethylene glycol (PEG) or other biocompatible polymers coated with liposomes to extend the half-life of antigens [131]. However, some studies have shown that the slow release of the short peptide vaccine promotes the secretion of pro-inflammatory cytokines, resulting in the persistence of T cells at the vaccination site, inhibiting their movement to the tumor [132].

Adjuvants targeting TLRs have shown great potential as cancer vaccines adjuvants. For example, polyinosinic–polycytidylic acid (poly I: C, a TLR3 agonist) is the most potent inducer of Th1-type responses, inducing a robust CTLs response when combined with vaccines [133]. Poly(I: C) has been evaluated in melanoma and glioma clinical trials [134]. Imiquimod (a TLR7 against) activates TLR7/8 to induce a Th1-type response, producing cytokines (TNF-*α*, IFN-*γ*, IL-12). Imiquimod is already approved to treat genital warts and basal cell carcinoma [135, 136], and several clinical trials have been conducted [137]. Furthermore, CpG (a TLR9 agonist) has been widely tested in clinical trials of therapeutic cancer vaccines for melanoma, breast, and glioblastoma [138, 139]. Another class of potential adjuvant is the STING agonists. STING is a signal transduction molecule located in the endoplasmic reticulum involved in innate immune response induced by the virus. Several STING activators are under preclinical or clinical study [140]. Additionally, cytokines such as IL-2, GM-CSF, and IFN have acquired promising results in clinical trials as cancer vaccines adjuvants. GM-CSF has been tested in clinical trials as a vaccine adjuvant for anti-tumor immunotherapy in prostate, skin, breast, and lung cancer [141]. Furthermore, scientists are using different adjuvants simultaneously to increase the anti-tumor response. For instance, the scientists used a combination of IL-2 and TLR7 agonists to enhance CD8^+^ T cell responses and anti-tumor effects [132].

### Nucleic acid-based cancer vaccines

Nucleic acid vaccines deliver genetic information encoding tumor antigens to the host to produce antigen proteins through normal physiological activities. Then, the expressed tumor antigens could induce immune killing effects against cancer cells. Because of the ubiquity of the RNA enzyme and the structural differences between DNA and mRNA, DNA has better stability and a long time of the presence in the body than mRNA. Thus, early nucleic acid vaccines primarily focused on DNA vaccines. DNA molecules need to enter the cell nucleus to initiate transcriptional, while mRNA enters the cytoplasm to translate and express antigens directly. Therefore, mRNA antigen production is instantaneous and efficient. DNA vaccines need an extra step to go into the cell nucleus, leading to a lower immune response than mRNA vaccines. However, once plasmid DNA enters the nucleus, a single plasmid DNA can produce multiple mRNA copies, producing more antigens than a single mRNA molecule. In addition, DNA vaccines also have a potential risk of insertion mutations, and mRNA have no risk of insertion and integration into the genome.

#### DNA vaccine

Cancer DNA vaccines are based on bacterial plasmids that encode one or several oncology antigens inducing innate immunity activation and adaptive immune responses [142]. As early as 1990, Wolff et al. directly injected naked DNA into murine muscle and observed the expression of corresponding proteins [143]. The first human trials of DNA vaccine to treat immunodeficiency virus type 1 (HIV) were reported in 1998 [144]. Although we have been studying DNA vaccines for a long time, there is still limited results. Until recently, India approved a COVID-19 DNA vaccine (ZycoV-D). ZycoV-D was approved as the world's first DNA vaccine for humans, heralding the arrival of DNA vaccines for various diseases.

DNA vaccines induce both humoral and cellular immune responses. DNA vaccines need to enter the nucleus to be transcribed and then translated to the encoded antigens in the cytoplasm. The antigen is processed and presented to CD8^+^ T and CD4^+^ T cells by MHC I and MHC II molecules to activate specific immune responses. The action modes of DNA vaccines can be divided into three categories [12]. DNA goes directly into a somatic cell, such as a muscle cell. After translation, the DNA-encoded antigens are directly delivered to cytotoxic CD8^+^T cells by MHC-1 molecules. The second pathway is that the antigen encoded by DNA in somatic cells is released by secreting or apoptotic bodies. These peptides are phagocytosed, processed by APCs and cross-presented by MHC II molecules to CD4^+^ T cells. The third pathway is directly transfecting DNA into APCs. The endogenous antigens produced by APCs are processed and presented to CD8^+^ T and CD4^+^ T cells by MHC I and MHC II, respectively [145]. The activation of CD4^+^ T cells induces humoral immunity. CD8^+^ T cells are differentiated into CTLs to induce cellular immunity. Direct transfection of DNA plasmids into APCs, mainly through intradermal delivery, is considered the most critical pathway for DNA cancer vaccines [145].

Moreover, CpG motifs in plasmid DNA help activate innate immune responses. CpG motifs can interact with TLR9 as a danger signal. TLR9 triggers a signaling cascade that activates NF-κB, IRAK and elicits the production of chemokines and inflammatory cytokines [146]. DNA double-stranded structure also activates the STING signaling pathway. STING is a primary DNA sensor that controls the cascade of cytoplasmic DNA signals independent of TLR, which is why DNA vaccines did not produce a robust adaptive immune response in STING deficient mice [147]. DNA vaccines can encode multiple antigens or large antigens. DNA vaccines are highly specific and safe, with lower production costs and they are facile to transport and store. The insertional mutation rate of DNA vaccines is lower than the spontaneous mutations rate and the DNA rarely binds to host chromosomes [148, 149]. Furthermore, the tumor antigen expressed by DNA cancer vaccines has the same species modification as the natural tumor antigen. DNA cancer vaccines have particular advantages, and optimized DNA vaccines have proven efficacy in preclinical models [150]. However, DNA vaccines have only achieved minor progress in clinical trials due to their poor immunogenicity [151].

##### Optimization strategies

There are several strategies to improve the immunogenicity of DNA vaccines. One of the strategies is the optimization of plasmid elements. For instance, the Kozak sequence before initiation codon, species-specific codons, and intron sequence should be considered [152]. A powerful promoter sequence is needed to allow efficient transcription. Modified strong viral CMV promoters have been proved to improve antigen expression efficiency [153]. Additionally, adjuvants are often used to enhance the immunogenicity of DNA vaccines, such as CpG motifs, polymer, nanoparticle, liposome, and small molecule agonists [145]. Finally, optimizing the design of tumor antigens is also crucial for improving DNA vaccines [154].

Chimeric antigen and multi-epitope antigen can effectively enhance the immunogenicity of DNA vaccines. Chimeric DNA vaccines encode xenoantigens. Xenoantigens are “non-autoantigens” that can bypass immune tolerance and have appropriate homology allowing cross-recognition by T cells [155]. Vaccines based on xenoantigens have been approved for use in treating animal tumors. For example, a DNA vaccine against human tyrosinase is approved to treat oral malignant melanoma in dogs [156]. Chimeric protein DNA cancer vaccines encoding heterologous and homologous antigens have also effectively enhanced immunogenicity [157]. Coding for two proteins where the heterologous part is responsible for bypassing immune tolerance and the homologous sequence stimulates the activation of specific immune responses is also viable [156]. DNA vaccine encoding human and mouse prostate-specific membrane antigen has been clinically studied (NCT00096629). In addition, the construction of a multi-epitope DNA vaccine is also a meaningful way to enhance immunogenicity. DNA vaccines containing multiple antigen genes can induce a wide range of CTLs responses specific to multiple antigens [158]. A recently preclinical study of multi-neoantigen DNA vaccine was revealed that the vaccine could induce predominant CD8^+^ T cell response in mouse tumor models [159]. Another strategy to elicit CD8^+^ T cell responses is to fuse antigens and chemokine for enhancing targeting to DCs [160, 161]. Multi-epitope DNA vaccine can overcome the mutation or loss of tumor antigen to a certain extent, which is a potential approach to address the tumor heterogeneity and immunogenicity loss associated with TAAs [158].

#### mRNA vaccines

Recently, the FDA approved two COVID-19 mRNA vaccines (Moderna′s Spikevax and Pfizer's BNT162b2). BNT162b2 is also the first mRNA vaccine approved for marketing by the FDA. The advantage of mRNA vaccines in rapid response to COVID-19 global outbreaks has led to a dramatic increase in the market value of the mRNA vaccines. Currently, CureVac, BioNTech and Moderna are the pioneers and leaders in the field of in vitro transcribed (IVT) mRNA vaccines. mRNA vaccine is a promising cancer vaccines platform, introducing exogenous synthetic mRNA into cells to provide antigen synthesis templates. Expressed antigens are delivered to the surface of APCs via MHC molecules to activate anti-tumor immunity [162, 163]. In 1990, scientists successfully expressed luciferase, beta-galactosidase, and chloramphenicol acetyltransferase in vivo, proving the mRNA vaccine's feasibility [143]. Jirikowski found that mRNAs encoding oxytocin and vasopressin injected into diabetes insipidus rats could observe a temporary reversal of their diabetes insipidus within hours of the injection in 1992 [164]. Nowadays, mRNA cancer vaccines have achieved some promising clinical responses in treating a variety of solid tumors [165]. Furthermore, it was found that the mRNA vaccine could enhance the efficacy of other therapies. For instance, an mRNA vaccine, encoding a chimeric receptor directed toward CLDN6 a target expressed on certain solid cancer, was proved to enhance the efficacy of claudin-CAR-T cells against solid tumors [166].

mRNA vaccines are mainly divided into non-replicating mRNA and self-amplifying RNA (SAM). Non-replacing mRNA is composed of 7-methylguanosine (m7G) 5′ cap, 5'-untranslated region (5'-UTR), open reading frame (OFR), 3'-untranslated region (3'-UTR), and 3'poly(A) tail [163]. These elements are important for mRNA stability and transcription factor recruitment, affecting the effective translation of proteins. The 5′cap and 3′poly(A) tail can be added by enzyme after the initial IVT. Unlike non-replacing mRNA, there are two OFRs in SAM. One encodes objective antigen, and another encodes viral replication component, enabling long-lasting RNA amplification in cells. SAM is originated from alphavirus, and it replicates and magnifies in vivo to induce a persistent and efficient immune response. SAM allows low doses of vaccination to produce large amounts of the antigen over a certain period [167]. However, the applications of SAM in cancer vaccines are still at the preclinical stage, and its clinical application needs to be further studied [168]. Cancer mRNA vaccines are mostly non-replicating [169, 170]. Thus, we primarily focus on non-replicating mRNA.

mRNA vaccines have several advantages. mRNA vaccines allow simultaneous encoding of multiple antigens and full-length tumor antigens. Encoding multiple antigens induces broader humoral and cellular immunity increasing the chances of overcoming resistance to cancer vaccines. Encoding full-field tumor antigens simultaneously and cross-presenting multiple epitopes of HLA by APCs could induce a broader T-cell response [171]. Furthermore, mRNA vaccine can be produced quickly, flexibly and efficiently [5]. Thus, mRNA is an ideal platform for personalized neoantigen vaccine preparation [169, 172]. IVT utilizes bacteriophage RNA polymerase and linearized DNA template to transcribe mRNA in vitro. IVT does not depend on cells and related complex regulations, making mRNA production simpler, faster, and clearer. Finally, mRNA vaccine has high safety since they will not be integrated into the host genome. mRNA vaccine effectively induces MHC I mediated CD8^+^ T cell responses, which is suitable for cancer treatment. Although mRNA vaccine has several advantages, its development is limited by its instability, innate immunogenicity and inefficiency of in vivo delivery [173–175].

##### Optimization strategies

In recent decades, mRNA's stability and translation efficiency have been improved by various modifications of the backbone and untranslated regions. New purification techniques such as fast protein liquid chromatography (FPLC) make mRNA products free of double strands, reducing non-specific immunity activation. Moreover, lipid nanoparticles (LNPs) and other new delivery technologies have greatly improved the efficiency of mRNA delivery in vivo. Stabilizing the mRNA is the key to ensuring its expression efficiency. Multiple strategies can be used to improve the stability of mRNA and translation efficiency. 5'cap is crucial for the efficient translation of mRNA into protein [176]. The 5′cap of IVT mRNA recruits the eukaryotic translation initiation factor 4E (EIF4E) to facilitate ribosome recognition and translation initiation, and it also prevents mRNA degradation by binding with decapping enzyme [177]. Abnormally capped and uncapped mRNAs will be recognized by the PRRs, such as MDA5 and RIG-I, activating type-1 IFN production to establish an antiviral state, blocking mRNA translation [178, 179]. Traditional enzyme and chemical capping methods are inefficient and expensive. Scientists recently developed an anti-reverse cap analog to overcome these shortcomings. The co-transcriptional capping method is called CleanCap™ [180].

The poly(A) sequence improves translation efficiency by increasing mRNA stability and slowing down exonuclease-mediated mRNA degradation. Poly(A)-binding proteins can directly interact with 5′cap-binding complex eIF4G to form a closed-loop state to promote mRNA stability and translation [181, 182]. Commonly used Poly(A) is 250 units in length, but the longer does not mean the better. For instance, a recent study found that shorter poly(A) sequence could promote efficient translation [183]. Additionally, optimizing UTRs increases mRNA stability and translation efficiency through interaction with a series of transcriptional factors. For instance, removing highly stable secondary structures in UTRs helps recruit ribosomes [5, 184]. In addition, codon optimization of the ORF also regulates translation efficiency. Appropriate GC content, replacing rare codons, and avoiding hairpin loops are be applied to improve translation efficiency [185].

Another critical factor limiting the development of mRNA vaccines is the activation of innate immunity. mRNA activates the innate immune response through various RNA sensors such as TLRs, RIG-I and PKR [186–189]. This immunostimulation may be beneficial for vaccines as an adjuvant. However, this immunostimulation can also hinder mRNA translation. Modifying mRNA transcripts with alternative nucleotides can effectively reduce innate immune activation, such as replacing cytidine with 5-methylcytidine(m5C), replacing uridine with pseudouridine (Ψ) or 1-methylpseudouridine (m1Ψ) [190, 191]. Besides, epigenomic modifications of post-transcriptional RNA also regulate mRNA translation efficiency and immunogenicity [192]. Furthermore, various pollutants such as dsRNA in mRNA may activate PPRs. High-purity mRNA contributes to minimal innate immune activation. HLPC is often used to scalable purity mRNA [193, 194]. Recently, some new methods of purification have been successfully used in the laboratory [195].

Various vectors have been used to improve the delivery efficiency of mRNA cancer vaccines, including several viral, non-viral, and cell-based vehicles [5, 196]. LNPs are the most widely used delivery system. LNPs are mainly composed of ionizable amino lipid-like molecules, phospholipids, cholesterol, as well as lipid-anchored PEG. Phospholipid and cholesterol primarily stabilize liposome structure and aid membrane fusion and in vivo escape. Lipid-anchored PEG helps prevent particle aggregation, improve storage stability and reduce macrophage-mediated clearance [197]. Ionizable lipids, the key component of the LNPs delivery and function [198], is the main target for LNPs improvement. Optimization of ionizable lipids focuses on regulating head groups, connectors, and alkyl chains to regulate acid dissociation constant (pKa), fusion properties and metabolic behavior. Representative ionizable lipids include dilinoleylmethyl-4-dimethylaminobutyrate (DLin-MC3-DMA) [199], 1,2-dioleoyl-sn-glycerol-3-phosphoethanolamine (DOPE) [200]. Furthermore, the ideal lipid material should be metabolized and cleared quickly after mRNA delivery, thus reducing the toxic side effects caused by the carrier and allowing multiple administrations [201]. The uptake pathways of LNPs mainly contain apolipoprotein or albumin receptor-mediated endocytosis and non-specific pinocytosis [202]. Recently, LNPs have been modified to achieve targeted delivery. For example, LNPs modified with mannose could target DCs through the mannose receptor CD206 [203]. Moreover, LNPs prepared by chip-based microfluidic devices have the advantages of high stability and repeatability, which is beneficial to GMP production of LNPs [203].

## Clinical application

### Cell-based cancer vaccines

The clinic trials of cell-based cancer vaccines that should be focused on recently contain two sorts: engineered tumor cell vaccine and DC vaccine. We summarize the clinical trials of cell-based cancer vaccines initiated in the last five years (Table 1). One engineered tumor cell vaccine representative is the GVAX vaccine, the tumor cell transfected with the GM-CSF gene. Through the expression of GM-CSF, the vaccine can increase anti-tumor efficacy by regulating the activity of many immune cells such as DCs and NK cells. In clinical trials, the effectiveness has been proved in different cancers [204, 205]. GVAX vaccine is currently assessed in clinical trials combined with other drugs to improve the anti-tumor efficacy further. Clinical trials of GVAX in combination with CRS-207, PD-1 or Urelumab are ongoing (NCT03190265, NCT02451982). The combination of cancer vaccines and ICs blockade has been an effective strategy for cancer treatment. By combining PD-1/CTLA-4 with the GVAX vaccine, both the CD8^+^/Treg ratios and IFN-*γ*^+^TNF-*α*^+^ CD8^+^ tumor-infiltrating lymphocytes increased significantly [206]. Besides, the gene modification which could modify the whole cancer cell to cancer stem cells-like cells was also used in the whole cancer cell vaccine. AGI-101H, a gene-modified melanoma stem cells-like vaccine, was proved to be able to treat patients with melanoma. In two phase II studies, the patients with melanoma seemed to benefit from AGI-101H. The vaccination contributed to their increased long-term survival of them [207].

**Table 1:** Selected ongoing clinical application of cell-based cancer vaccines (2016–2021)

NCT Number	Status	Conditions	Phases	category
NCT03190265	Recruiting	Pancreatic Cancer	II	Tumor cell
NCT02648282	Active, not recruiting	Pancreatic Cancer	II	Tumor cell
NCT02451982	Recruiting	Pancreatic Cancer	I/II	Tumor cell
NCT03767582	Recruiting	Advanced Pancreatic, Ductal Adenocarcinomas	I/II	Tumor cell
NCT03161379	Active, not recruiting	Pancreatic Cancer	II	Tumor cell
NCT02194751	Not yet recruiting	Follicular Lymphoma	II	Tumor cell
NCT03376477	Recruiting	Multiple Myeloma	II	Tumor cell
NCT03328026	Recruiting	Breast Cancer Female, Breast Neoplasm Female	I/II	Tumor cell
NCT03096093	Recruiting	Cancer, Neoplasms	I/II	Allogeneic cell
NCT03970746	Recruiting	Non-Small Cell Lung Cancer	I/II	DC
NCT03879512	Recruiting	Childhood Glioblastoma	I/II	DC
NCT03059485	Recruiting	Acute Myelogenous Leukemia	II	DC
NCT04912765	Recruiting	Hepatocellular Cancer, Colorectal Cancer, Liver Metastases	II	DC
NCT02919644	Recruiting	Colorectal Cancer, Curative Resection	II	DC
NCT04166006	Recruiting	Head Neck Tumors, Neuroendocrine Tumors, Soft Tissue Sarcoma, Rare Cancer	II	DC
NCT03406715	Active, not recruiting	Small Cell Lung Cancer, Lung Cancer	II	DC
NCT04523688	Not yet recruiting	Glioblastoma	II	DC
NCT04567069	Recruiting	Gastric Cancer	I/II	DC
NCT04388033	Recruiting	Glioblastoma, Neuroepithelial, Neuroectodermal Tumors, Neoplasms	I/II	DC
NCT04487756	Recruiting	Extensive-stage Small Cell Lung Cancer	I/II	DC
NCT02649829	Recruiting	Malignant Pleural Mesothelioma	I/II	DC
NCT03395587	Recruiting	Glioblastoma	II	DC
NCT03548571	Recruiting	Glioblastoma	II/III	DC
NCT04277221	Recruiting	Glioblastoma Multiforme	III	DC
NCT04317248	Recruiting	Hepatocellular Carcinoma	II	DC
NCT03035331	Recruiting	Non-Hodgkin Lymphoma, Small Lymphocytic Lymphoma	I/II	DC
NCT02465268	Recruiting	Glioblastoma Multiforme, Glioblastoma, Malignant Glioma, Astrocytoma	II	DC
NCT03400917	Active, not recruiting	Newly Diagnosed Glioblastoma	II	DC
NCT03384914	Recruiting	Breast Cancer	II	DC

The clinical trials have shown many positive results in terms of DC vaccines. For instance, a phase IIb trial of tumor lysate, particle-loaded, dendritic cell vaccine demonstrated a significantly increased disease-free survival compared the vaccine arm (62.9%) to the placebo arm (34.8%) in per treatment analysis in the treatment of melanoma [208]. In addition, DC vaccines are now known as one of the effective ways to break through the bottleneck of glioma treatment. A phase III clinical trial that evaluated the effectiveness of a DC vaccine to glioblastoma has already shown promising results. The addition of the DC vaccine named DCVax‐L significantly extends patients' median overall survival (n = 331) to 23 months [209]. Furthermore, the chemotherapeutic drugs could also induce the release of danger signals of tumor cells like damage-associated molecular pattern molecules (DAMP), which can regulate DC cells [210, 211]. Hence, it is predictable that adjuvant chemotherapy could improve DC vaccines, proved in both mouse models and clinic trials [212]. The contribution of chemotherapy to cancer vaccines is complex, and it may also include the restraining of immunosuppression [211].

### Virus-based cancer vaccines

Virus-based cancer vaccines have been used for anti-tumor applications (Table2), particularly adeno-associated viruses. Adenovirus-based vaccines that provide TAAs or carry immune-stimulating genes have been shown to induce strong anti-tumor immunity in preclinical and clinical trials. VRP-HER2, a viral vector-based vaccine encoding HER2, has shown encouraging results in preclinical and clinical trials. After the VRP-HER2 vaccination, the hHER2 + breast cancer mice model illustrated an improvement in tumor progression. A similar benefit of VRP-HER2 to breast cancer has also been found in the clinical trial. The lengthened progression-free of patients is tightly related to the HER2-specific immune response [213]. Furtherly, anti-PD-1 therapy was tried to combine with VRP-HER2 to improve the overall efficacy. The idea was tentatively proved to be feasible [214]. And the clinical trial is still ongoing (NCT03632941). Nadofaragene firadenovec, a non-replicating adenovirus vector vaccine encoding human IFN-*α*, has shown good potential in treating non-muscular-invasive bladder cancer. The results from the clinical trial, which tested the effectiveness of Nadofaragene firadenovec to BCG-unresponsive non-muscular-invasive bladder cancer, have shown that 53.4% of patients have a complete response after the first dose [215].

**Table 2:** Selected ongoing clinical application of virus-based cancer vaccines (2016–2021)

NCT number	Status	Targeted antigens/biological	Conditions	Phases	Category
NCT03136406	Active, not recruiting	mutant KRAS	Pancreatic Cancer	I/II	Virus vector
NCT03329248	Active, not recruiting	ALT-803, ETBX-011, GI-4000	Pancreatic Cancer	I/II	Virus vector
NCT03632941	Recruiting	HER2	Breast Cancer	II	Virus vector
NCT04410874	Recruiting	MUSIC-01	Non-melanoma Skin Cancer, Squamous /Basal Cell Carcinoma	I/II	Virus vector
NCT04432597	Recruiting	PRGN-2009	HPV-associated Cancers	I/II	Virus vector
NCT03815942	Active, not recruiting	ChAdOx1-MVA 5T4	Prostate Cancer	I/II	Virus vector
NCT03547999	Active, not recruiting	MVA-BN-CV301	Metastatic Colorectal Cancer	II	Virus vector
NCT03113487	Recruiting	p53	Recurrent Ovarian, Primary Peritoneal, Fallopian Tube Cancer	II	Virus vector
NCT04111172	Recruiting	Ad5.F35-hGCC-PADRE	Gastrointestinal Adenocarcinoma	II	Virus vector
NCT03315871	Recruiting	PROSTVAC-V/F, CV301	Prostate Cancer	II	Virus vector
NCT04574583	Active, not recruiting	MVA-BN-CV301	Metastatic Cancer, Solid Tumors	I/II	Virus vector
NCT02649855	Active, not recruiting	PROSTVAC-F/V	Prostate Cancer, Prostate Neoplasms, Neoplasms, Prostatic	II	Virus vector
NCT02933255	Recruiting	PROSTVAC-V/F	Prostate Cancer	I/II	Virus vector
NCT03563157	Active, not recruiting	MUC1, HER2, IL-15	Colorectal Cancer Metastatic, mCRC	I/II	Virus vector
NCT03953235	Recruiting	Neoantigens	Non-Small Cell Lung Cancer, Colorectal Cancer, Pancreatic Cancer, Solid Tumor	I/II	Virus vector/mRNA

Furthermore, BT-001, an oncolytic virus vaccine, which could express anti-CTLA4 antibody and GM-CSF, is worthy of being concentrated on. It has already been tested in a clinical trial (NCT04725331). Another oncolytic virus vaccine named T-VEC was used in a phase II study. The objective/complete response rates were32%/18% in patients with stage IIIB-IVM1a disease and 28%/14% (112 participants) in the overall population [216]. This study showed that T-VEC provoked systemic immune activity and changed the tumor microenvironment, which may enhance the effectiveness of other immunotherapy agents in combination therapy.

### Peptide-based cancer vaccines

As early as 1996, Hu et al. reported the clinical trial results of peptide vaccine based on MAGE-1 [217]. Now various peptide-based cancer vaccines have been used to treat many types of cancer, including lung cancer [218], melanoma [24], pancreatic cancer [219], esophageal cancer, squamous carcinoma of the head and neck [220]. We reviewed clinical trials of peptide-based cancer vaccines launched in the last five years (Table 3). Peptide-based cancer vaccines in clinical trials typically contain multiple targets or epitopes to activate T cells that recognize different targets to minimize tumor-immune escape due to antigen loss. IMU-131 is a polypeptide fused to diphtheria toxin by B cell epitopes in the HER2 extracellular domain. A published phase I clinical trial results showed that IMU-131 induced HER2-specific antibodies and cellular responses [221]. NeuVax, another peptide vaccine targeting HER2, is one of the cancer vaccines to enter phase III clinical trials. Unfortunately, a recently released Phase III clinical trial results show that NeuVax alone has no significant effect on breast cancer [222]. Additionally, two phases II clinical evaluating NeuVax in combination with Trastuzumab for HER2-positive breast cancer are ongoing (NCT02297898 and NCT01570038).

**Table 3:** Selected ongoing clinical application of peptide-based cancer vaccines (2016–2021)

NCT number	Status	Targeted antigens/biological	Conditions	Phases	Category
NCT04747002	Recruiting	DSP-7888	Acute Myeloid Leukemia in Remission	II	Peptide
NCT03946358	Recruiting	UCPVax	Squamous Cell Carcinoma of the Head and Neck, Anal Canal Cancer, Cervical Cancer	II	Peptide
NCT04263051	Recruiting	UCPVax	Advanced Non-small Cell Lung Cancer	II	Peptide
NCT04382664	Recruiting	UV1	Malignant Melanoma	II	Peptide
NCT04114825	Active, not recruiting	RV001V	Prostate Cancer Recurrent	II	Peptide
NCT02938442	Recruiting	P10s-PADR	Triple Negative Breast Cancer, Breast Neoplasms	I/II	Peptide
NCT04280848	Recruiting	Telomerase	Glioblastoma	I/II	Peptide
NCT02818426	Recruiting	UCPVax	Metastatic Non-small Cell Lung Cancer	I/II	Peptide
NCT04369937	Recruiting	HPV-16 E6/E7	HPV-Related Squamous Cell Carcinoma, Head and Neck Squamous Cell Carcinoma	II	Peptide
NCT02654587	Active, not recruiting	OSE2101	Non-Small Cell Lung Cancer	III	Peptide
NCT03149003	Recruiting	DSP-7888	Glioblastoma	III	Peptide
NCT04024800	Active, not recruiting	AE37 Peptide	Triple-negative Breast Cancer	II	Peptide
NCT02865135	Active, not recruiting	DPX-E7	Cancer of Head and Neck, Cancer of Cervix, Cancer of Anus	I/II	Peptide
NCT03311334	Recruiting	DSP-7888	Renal Cell Carcinoma, Urothelial Carcinoma, Primary Peritoneal Cancer, Ovarian Cancer,	I/II	Peptide
NCT03012100	Recruiting	Folate Receptor-*α*	Breast Cancer	II	Peptide
NCT03606967	Recruiting	Neoantigens	Breast Carcinoma	II	Peptide
NCT04580771	Recruiting	HPV-16 E6/E7	Cervical Cancer	II	Peptide
NCT04206254	Not yet recruiting	gp96	Liver Cancer	II/III	Peptide
NCT03029403	Recruiting	DPX-Survivac	Advanced Cancer, Ovarian Cancer, Primary Peritoneal Carcinoma, Fallopian Tube Cancer	II	Peptide
NCT02636582	Active, not recruiting	NeuVax	Breast Ductal Carcinoma in Situ	II	Peptide
NCT03715985	Recruiting	Neoantigens	Malignant Melanoma, Non-Small Cell Lung Cancer Metastatic, Bladder Urothelial Carcinoma, Metastatic	I/II	Peptide
NCT04197687	Recruiting	Multi-epitope HER2 Peptide	Breast Cancer	II	Peptide
NCT03560752	Recruiting	CMV-MVA Triplex	CMV Viremia in Participants with Blood Cancer Undergoing Donor Stem Cell Transplant	II	Peptide
NCT04300244	Recruiting	UV1 vaccine	Malignant Mesothelioma	II	Peptide
NCT04106115	Not yet recruiting	S-488210/S-488211	Bladder Cancer	I/II	Peptide
NCT03559413	Active, not recruiting	Neoantigens	Primary, Relapsed Acute Lymphoblastic Leukemia of Childhood, Adolescents and Young Adults	I/II	Peptide
NCT03258008	Active, not recruiting	ISA101b	Oropharyngeal Cancer	II	Peptide
NCT04051307	Recruiting	Arginase1, PD-L1	Polycythemia Vera, Essential Thrombocythemia	I/II	Peptide
NCT04646005	Recruiting	ISA101b	Cervical Cancer	II	Peptide
NCT03821272	Recruiting	PepCan	Head and Neck Cancer	I/II	Peptide
NCT03761914	Recruiting	WT1	Acute Myelogenous Leukemia, Ovarian Cancer, Colorectal Cancer, Triple-negative Breast Cancer, Small-cell Lung Cancer	I/II	Peptide
NCT04116658	Recruiting	EO2401	Glioblastoma	I/II	Peptide
NCT02960230	Recruiting	H3.3K27M Peptide	Diffuse Intrinsic Pontine Glioma, Glioma, Diffuse Midline Glioma, H3 K27M-Mutant	I/II	Peptide
NCT03018288	Active, not recruiting	HSPPC-96	Glioblastoma	II	Peptide
NCT02795988	Active, not recruiting	IMU-131	Gastrointestinal Neoplasms, Adenocarcinoma	I/II	Peptide
NCT02785250	Active, not recruiting	DPX-Survivac	Recurrent Epithelial Ovarian Cancer, Recurrent Fallopian Tube Cancer, Recurrent Peritoneal Cancer	I/II	Peptide
NCT04060277	Recruiting	Triplex	Leukemia, Lymphoma	II	Peptide
NCT03848039	Not yet recruiting	Gardasil-9	Cervical Intraepithelial Neoplasia	III	Protein
NCT03979014	Not yet recruiting	GARDASIL9	Neoplasia	III	Protein
NCT02955290	Active, not recruiting	CIMAvax	Non-small Cell Lung Cancer, Squamous Head and Neck Cancer	I/II	Protein
NCT04910802	Recruiting	Gardasil9	HPV Infection, CIN 2, 3, Cervical Cancer	IV	Protein
NCT03284866	Recruiting	Gardasil 9	AIDS-Related Human Papillomavirus Infection, High Grade Cervical Squamous Intraepithelial Neoplasia, HIV Infection	III	Protein
NCT03206047	Active, not recruiting	CDX-1401	Recurrent Ovarian, Fallopian Tube, or Primary Peritoneal	I/II	Protein
NCT03728881	Active, not recruiting	Cervarix, Gardasil	Human Papillomavirus-Related Cervical Carcinoma	III	Protein
NCT02568566	Active, not recruiting	Gardasil 9	Human Papillomavirus-Related Carcinoma	II	Protein
NCT03180034	Active, not recruiting	Gardasil, DTaP, Cervarix	Human Papillomavirus-Related Cervical Carcinoma	IV	Protein
NCT04635423	Recruiting	V503	Warts, Genital, Neoplasms, Anal	III	Protein
NCT04782895	Active, not recruiting	Gardasil®9	Cervical Cancer, Condylomata Acuminata	III	Protein
NCT02834637	Active, not recruiting	Cervarix, Gardasil 9	Human Papilloma Virus	III	Protein
NCT04508309	Recruiting	Gardasil®, Cecolin®	Cervical Cancer	III	Protein
NCT03675256	Active, not recruiting	Gardasil 9, Cervarix	Papillomavirus Infections	IV	Protein
NCT03036930	Active, not recruiting	Gardasil 9	Human Papillomavirus Infection	II	Protein
NCT04274153	Recruiting	Gardasil9	Immunization, Human Papilloma Virus	IV	Protein
NCT03702231	Active, not recruiting	Zoster Vaccine Recombinant	Chronic Lymphocytic Leukemia, Small Lymphocytic Lymphoma	II	Protein
NCT04459221	Recruiting	Gardasil 9	Papilloma Viral Infection	IV	Protein
NCT03579654	Not yet recruiting	PSA, IL-2, GM-CSF	Prostate Cancer	II	Protein

DSP-788 is another cancer polypeptide vaccine developed by Boston Biomedical. DSP-7888 contains peptides that induce Wilm tumor gene1 (WT1)-specific CTLs and helper T cells. Hence, it can logically attack WT1-expressing cancer cells in various hematological and solid tumors. DSP-7888 is well tolerated in patients with recurrent or advanced malignancies and has no dose-limiting toxicity [223]. Three phases I/II trials have evaluated the safety and efficacy of DSP-7888 as a single agent, and clinical data have not been published yet(NCT02436252, NCT02750891, NCT02498665). Furthermore, DSP-7888 is also under evaluation in a clinical Phase II trials treating acute leukemia patients (NCT04747002) and a Phase III clinical trial targeting patients with recurrent glioblastoma in combination with Bevacizumab (NCT03149003). SurVaxM is another promising peptide vaccine against glioblastoma that significantly prolonged the survival of patients with glioblastoma [224]. SurVaxM in combination with Pembrolizumab for glioblastoma is evaluated in phase II clinical trial (NCT04013672). Furthermore, neoantigen personalized vaccine has been the focus of recent years. A recently published Phase I clinical trial showed that melanoma patients who received a personalized peptide cancer vaccine still had a durable anti-tumor response and effective cancer control after four years [24].

### DNA Vaccine

Multiple DNA cancer vaccines have undergone preclinical and clinical studies in the past decade. Nevertheless, only a small number of clinical trials have published the results. Most DNA vaccines in clinical trials are based on TAAs initially. This trend has changed and the DNA vaccine is dominated by personalized vaccines that encode neoantigens. We reviewed clinical trials of DNA-based cancer vaccines initiated in the last five years (Table 4). The DNA vaccine has been mostly studied in cervical cancer. VGX-3100, a DNA vaccine against HPV, is currently evaluated for safety and efficacy in two Phase III clinical trials (NCT03185013, NCT03721978). Recently, INOVIO company announced positive results from the first phase III trial of VGX-3100 for cervical precancerous lesions (NCT03185013). VGX-3100 is the first DNA drug in the world that is promising to market in addition to the COVID-19 vaccine "ZycoV-D."

**Table 4:** Selected ongoing clinical application of nucleic acid-based cancer vaccines (2016–2021)

NCT number	Status	Targeted antigens/biological	Conditions	Phases	Category
NCT04090528	Recruiting	pTVG-HP, pTVG-AR	Prostate Cancer, Metastatic Cancer	II	DNA
NCT03439085	Recruiting	VGX-3100	Human Papillomavirus Associated Cancers	II	DNA
NCT03600350	Recruiting	pTGV-HP	Prostate Cancer	II	DNA
NCT03603808	Recruiting	VGX-3100	HIV-Positive High-Grade Anal Lesions	II	DNA
NCT03444376	Recruiting	GX188E	Cervical Cancer	I/II	DNA
NCT04405349	Recruiting	VB10.16	Cervical Cancer, Cervix Cancer	II	DNA
NCT03548467	Active, not recruiting	VB10.NEO	Locally Advanced or Metastatic Solid tumors	I/II	DNA
NCT02780401	Active, not recruiting	HER2, IGFBP2, IFG-1R	Breast Cancer	I	DNA
NCT03199040	Active, not recruiting	Neoantigens	Breast Cancer	I	DNA
NCT03532217	Active, not recruiting	Neoantigens	Metastatic Hormone-Sensitive Prostate Cancer	I	DNA
NCT03721978	Recruiting	VGX-3100	Cervical Dysplasia, Cervical High Grade Squamous Intraepithelial Lesion	III	DNA
NCT03502785	Active, not recruiting	INO-9012	Urothelial Carcinoma	I/II	DNA
NCT03655756	Active, not recruiting	Emm55 streptococcal antigen	Cutaneous Melanoma	Early Phase I	DNA
NCT03491683	Active, not recruiting	WT1, PSMA, hTERT	Glioblastoma	I/II	DNA
NCT03122106	Active, not recruiting	Neoantigens	Pancreatic Cancer, Cancer of the Pancreas	I	DNA
NCT03897881	Recruiting	Neoantigens	Melanoma	II	mRNA
NCT03639714	Active, not recruiting	Neoantigens	Non-Small Cell Lung Cancer, Colorectal Cancer, Gastroesophageal	I/II	mRNA
NCT04382898	Recruiting	W-pro1 (BNT112)	Prostate Cancer	I/II	mRNA
NCT04534205	Recruiting	BNT113	Head and Neck Cancer	II	mRNA
NCT04526899	Recruiting	NY-ESO-1, MAGE-A3, Tyrosinase, TPTE	Melanoma Stage III/IV	II	mRNA
NCT03164772	Active, not recruiting	NY-ESO-1, MAGEC1, MAGE-C2, 5 T4, Surviving, MUC1	Metastatic Non-small Cell Lung Cancer, NSCLC	I/II	mRNA
NCT04163094	Recruiting	W-ova1	Ovarian Cancer	I	mRNA
NCT03468244	Recruiting	Neoantigens	Digestive System Cancer	Not Applicable	mRNA
NCT03313778	Recruiting	Neoantigens	Solid Tumors	I	mRNA
NCT03948763	Recruiting	KRAS mutations	Neoplasms, NSCLC, Pancreatic Neoplasms, Colorectal Neoplasms	I	mRNA
NCT03289962	Recruiting	Neoantigens	Melanoma, Non-Small Cell Lung	I	mRNA
NCT03815058	Recruiting	Neoantigens	Advanced Melanoma	II	mRNA
NCT04486378	Recruiting	Neoantigens	Colorectal Cancer	II	mRNA
NCT04161755	Recruiting	Neoantigens	Pancreatic Cancer	I	mRNA
NCT03788083	Recruiting	Trimix mRNA (mRNA encoding CD40L, CD70, acTLR4)	Breast Cancer Female, Early-stage Breast Cancer	I	mRNA
NCT03323398	Active, not recruiting	mRNA-2416 (mRNA encoding OX40L)	Relapsed/Refractory Solid Tumor Malignancies or Lymphoma, Ovarian Cancer	I/II	mRNA
NCT03739931	Recruiting	mRNA-2752 (mRNA encoding OX40L, IL-23, IL-36Ƴ)	Relapsed/Refractory Solid Tumor Malignancies or Lymphoma, Other Solid Tumor	I	mRNA
NCT03289962	Active, not recruiting	Neoantigens	Locally Advanced or Metastatic Tumors	I	mRNA

GX-188E is another DNA vaccine for cervical cancer that fuses multiple epitopes. GX-188E has a specific targeting ability to activate dendritic cells. In a phase II trial of GX-188E for cervical cancer, 67% of 52 patients treated with GX-188E had reduced lesions after 36 weeks [225]. The clinic trials of the combination therapy involved GX-188E have shown a bright perspective. GX-188E showed a remarkable overall response rate of 42% for the treatment of advanced cervical cancer combined with a PD-1 antibody called Pembrolizumab. Compared to using Pembrolizumab alone, the therapeutic effect improved significantly [226, 227]. Furthermore, the results from a phase I clinical trial of a personalized DNA vaccine for multiple myeloma are encouraging. Overall survival was 64% after a median follow-up of 85.6 months [228]. DNA cancer vaccines have also shown safety and tolerability in initial clinical trials treating multiple prostate and breast cancers [229, 230].

### mRNA vaccines

Several cancer vaccines based on IVT mRNA have shown promising results in clinical trials. The mRNA vaccines have been used to treat aggressive and metastatic solid tumors such as colorectal cancer, melanoma, and non-small cell lung cancer. We summarized clinical trials of mRNA-based cancer vaccines initiated in the last five years (Table 4). The antigens encoded by mRNA can be divided into three categories: Immunostimulants, TAAs, and tumor neoantigens. mRNA vaccines encoding immunostimulants were mostly in situ cancer vaccines. Instead of coding antigens, immunostimulants induce tumor cell death and release tumor antigens through intratumor injection. Immunostimulants are often used with other vaccines or immunotherapies.

TriMix mRNA comprises three mRNA encoding CD70, CD40L and a constitutively active form of TLR4, respectively. TriMix mRNA has shown good tolerability and immunogenicity in several clinical trials [231]. Two recently published Phase II clinical trials showed that TriMix and tumor-associated antigen mRNA vaccines produced vigorous CD8^+^ T cell responses in patients with stage III or IV melanoma, showing favorable tumor response rates [232, 233]. Another representative of immunostimulants mRNA vaccine is mRNA-252. mRNA-2752 is a lipid nanoparticle encapsulating mRNAs encoding human OX40L, IL-23 and IL-36*γ*. mRNA-252, developed by Moderna to treat lymphoma. It is currently enrolled in a clinical trial (NCT03739931). Furthermore, mRNA vaccine mixtures encoding multiple TAAs have been used to treat metastatic melanoma in several clinical trials. For instance, BNT111 is an mRNA vaccine that encodes four TAAs (NY-ESO-1, MAGE-A3, tyrosinase, and TPTE). BNT111 is an effective immunotherapy for melanoma patients with CPI and demonstrates the universal utility of non-mutated shared tumor antigens as cancer vaccination targets [234]. BNT111 is also used with other immunotherapies for melanoma (NCT04526899).

mRNA vaccines that encode neoantigens have become the leader in personalized vaccines. The safety and tolerability of personalized mRNA cancer vaccines have been demonstrated in clinical trials [235, 236]. Personalized mRNA vaccines led by BioNTech and Moderna have shown promising anti-tumor effects in clinical trials [25]. For example, mRNA-4157, a personalized neoantigen cancer vaccine encapsulated in LNPs that encodes up to 34 neoantigens, was developed by Moderna. mRNA-4157, alone or combined with Pembrolizumab, has shown good anti-tumor efficacy in phase I trials in patients with unresectable solid tumors. mRNA-4157 is now in phase II clinical trials. In addition, BNT122 is a neoantigen cancer vaccine developed by BioNTech based on the mRNA personalized cancer vaccines platform. BNT122 encodes up to 20 patient-specific neoantigens. The previous Phase 1a/1b trial evaluated BNT122 monotherapy or in combination with the anti-PD-L1 antibody Atezolizumab in treating patients with solid tumors (NCT03289962). Experimental data show that BNT122 induces a neoantigen-specific T cell response and objective responses in clinical. Recently, a phase II clinical trial of BNT122 for the treatment of colorectal cancer is currently underway (NCT04486378). Additionally, we summarized some representative clinical trials with reported results in Table 5.

**Table 5:** Selected completed clinical application of cancer vaccines (2016–2021)

NCT number	Targeted antigens/biological	Results	Phases
NCT01302496	CD70, CD40 ligand, tyrosinase, gp100, MAGE-A3, or MAGE-C2	After more than five years, TriMixDC-MEL ipilimumab treatment resulted in median overall survival of 28% and a median progression-free survival of 18%. After a median follow-up of 390 weeks, 11 /39 patients are alive of whom seven remain free-from progression and have remained in complete remission following treatment with TriMIxDC-MEL ipilimumab	II
NCT02139267	HPV	52% (33/64) of patients at per-protocol analysis and 67% (35/52) of patients at extension analysis presented histopathologic regression after receiving the GX-188E injection. 73% (per-protocol study) and 77% (extension analysis) of the patients with histologic regression showed HPV clearance	II
NCT03481816	PSA, brachyury, MUC1	The primary endpoints were met and there were no DLTs. Seventeen of 17(100%) patients mounted T-cell responses to at least one TAA, whereas 8 (47%) of 17 patients mounted immune responses to all three TAAs	I
NCT03384316	MUC1, CEA	The vaccine was generally well-tolerated. Antigen-specific T cells to MUC1, CEA, and brachyury were generated in all patients (Ten patients enrolled on trial)	I
NCT03199872	RV001V	The vaccine was generally well-tolerated, Targeting of RhoC induced a potent and long-lasting T cell immunity in the majority of the patients (18 of 21 evaluable)	I/II
NCT02981524	GVAX	Seventeen patients were enrolled. Grade ≥ 3 treatment-related adverse events were observed in two patients. There were no objective responses, and the disease control rate was 18% by RECIST 1.1. Biochemical responses (≥ 30% decline in CEA) were observed in 7/17 (41%) of patients	II
NCT02179515	MVA-brachyury- TRICOM	One transient grade 3 adverse event occurs. Including all DLs and all cancer types, 28/34 (82%) patients developed brachyury-specific CD4 + and/or CD8 + T-cell responses after vaccination	I
NCT02153918	PROSTVAC-V/F	The treatment course was well-tolerated, with no serious adverse events (AEs) or toxicities > grade 2 attributed to the vaccine. A total of 13/25 patients (52%) developed peripheral T-cell responses to any of the three tested TAAs (non-neoantigens)	II
NCT02004262	GVAX, CRS-207	All treatments were generally well-tolerated. The study did not meet its primary efficacy endpoint. The combination of Cy/GVAX + CRS-207 did not improve survival over chemotherapy	II
NCT01876212	TBVA	All treatments were generally well-tolerated. 6 patients (13 evaluable patients) developed specific peripheral blood T cell responses against ≥ 3 vaccine-associated peptides, with further evidence of epitope spreading	II
NCT01867086	GMCSF, TGFβ	There were no treatment-related serious adverse events. FANG vaccine elicited an immune response correlating with prolonged survival	II
NCT01706458	PAP	The treatment course was well-tolerated, with no serious adverse events (AEs) or toxicities > grade 2 attributed to the vaccine. Median time to progression was less than 6 months and not statistically different between study arms. Th1-biased PAP-specific T-cell responses were detected in 11 individuals (completed treatments) and were not statistically different between study arms	II
NCT01570036	HER2	275 patients were randomized, there were no clinicopathologic differences between vaccine and control group	II
NCT01519817	Brachyury	The treatment was well-tolerated, with no DLTs. 17/31 (54%) (including all dose levels) of patients developed brachyury-specific CD4 and/or CD8 T-cell responses post-vaccination	I
NCT01417000	GVAX, CRS-207	The treatment was well-tolerated. Heterologous prime/boost with Cy/GVAX and CRS-207 extended survival for patients with pancreatic cancer	II
NCT01341652	PAP	Two-year metastasis-free survival was not different between study arms (41.8% vaccine v 42.3%; *P* = .97)	II
NCT00088413	MUC-1, CEA	Some patients who had limited tumor burden with minimal prior chemotherapy seemed to benefit from the vaccine	I/II

## Conclusions and perspectives

The development of cancer vaccines is an important breakthrough in treating solid tumors. In this review, we have summarized the working mechanisms, optimization strategies, and clinical progress of cancer vaccines, which might hopefully facilitate the future design of the cancer vaccines. In addition, we also highlighted the existing barriers for the cancer vaccine translation, such as tumor resistance, therefore, proposing a combination therapy to improve the clinical efficacy. With further understanding of the immunological mechanisms and sequencing technology development, personalized cancer vaccines might be rapidly developed. Personalized neoantigens could elicit an actual tumor-specific T cell response with limited central immune tolerance. However, eliminating tumor cells expressing a specific neoantigen lead to the outgrowth of tumor cells without the neoantigen [237]. Targeting multiple neoantigens within a single vaccine might be a direction to reduce immune evasion and effectively eliminate tumors. Highly effective neoantigens need to be further predicted and identified for that only a few neoantigens could induce effective anti-tumor-immune responses currently.

Additionally, the therapeutic trial objects of cancer vaccines are mainly tumor patients who have failed traditional treatment methods and progressed. Theoretically, cancer vaccine therapy is more suitable for patients with a complete immune system, a smaller tumor load and a greater risk of recurrence. Therefore, future clinical trials of cancer vaccines should fully consider the patient's immune system function and tumor burden. In conclusion, cancer vaccines are promising immune-therapeutics for stimulating immune system to kill tumors and establishing immune surveillance. However, much work remains to be done on identifying neoantigens, developing combination therapy, and optimizing vaccine platforms before cancer vaccines become a potent strategy in immunotherapy.

## Data Availability

Not applicable.

## Abbreviations

TAAs: Tumor-associated antigens
TSAs: Tumor-specific antigens
DCs: Dendritic cells
HBV: Hepatitis B virus
HPV: Human papillomavirus
HER2: Human epidermal growth factor receptor 2
PSA: Prostate-specific antigen
HLA: Human leukocyte antigen
MHC: Major histocompatibility complex
APCs: Antigen presenting cells
TME: Tumor microenvironment
IFN-*γ*: Interferon-*γ*
ADCC: Antibody-dependent cellular cytotoxicity
CTLs: Cytotoxic T lymphocytes
FasL: Fas ligand
TNF-*α*: Tumor necrosis factor α
MDSCs: Myeloid-derived suppressor cells
TAMs: Tumor-associated macrophages
CAFs: Cancer-associated fibroblasts
Tregs: T regulatory cells
VLPs: Virus-like particles
MoDCs: Monocyte-derived dendritic cells
DCexos: Exosomes-released dendritic cells
ICD: Immunogenic cell death
STING: Stimulator of interferon genes
CPI: Immune checkpoint inhibitors
PD-L1: Programmed Cell Death Ligand 1
CTLA-4: Cytotoxic T-lymphocyte-associated protein 4
cDCs: Conventional DCs
mDCs: Myeloid DCs
RT: Radiotherapy
TLRs: Toll-like receptors
ROS: Reactive oxygen species
GM-CSF: Granulocyte-macrophage colony-stimulating factor
TGF-*β*: Tissue growth factor *β*
HSP: Heat shock proteins
MAGE-1: Melanoma antigen1
MUC1: Mucin 1
PRRs: Pattern recognition receptors
HBcAg: Hepatitis B virus surface antigen
PEG: Polyethylene glycol
Poly(I:C): Polyinosinic–polycytidylic acid
IVT: In vitro transcribed
SAM: Self-amplifying RNA
5′-UTR: 5′-Untranslated region
OFR: Open reading frame
3′-UTR: 3′-Untranslated region
LNPs: Lipid nanoparticles
ICs: Immune checkpoints
